# Protein Oxidation Implicated as the Primary Determinant of Bacterial Radioresistance 

**DOI:** 10.1371/journal.pbio.0050092

**Published:** 2007-03-20

**Authors:** Michael J Daly, Elena K Gaidamakova, Vera Y Matrosova, Alexander Vasilenko, Min Zhai, Richard D Leapman, Barry Lai, Bruce Ravel, Shu-Mei W Li, Kenneth M Kemner, James K Fredrickson

**Affiliations:** 1 Department of Pathology, Uniformed Services University of the Health Sciences, Bethesda, Maryland, United States of America; 2 National Institute of Biomedical Imaging and Bioengineering, National Institutes of Health, Bethesda, Maryland, United States of America; 3 Biosciences Division and Advanced Photon Source, Argonne National Laboratory, Argonne, Illinois, United States of America; 4 Biological Sciences Division, Pacific Northwest National Laboratory, Richland, Washington, United States of America; Brandeis University, United States of America

## Abstract

In the hierarchy of cellular targets damaged by ionizing radiation (IR), classical models of radiation toxicity place DNA at the top. Yet, many prokaryotes are killed by doses of IR that cause little DNA damage. Here we have probed the nature of Mn-facilitated IR resistance in *Deinococcus radiodurans,* which together with other extremely IR-resistant bacteria have high intracellular Mn/Fe concentration ratios compared to IR-sensitive bacteria. For in vitro and in vivo irradiation, we demonstrate a mechanistic link between Mn(II) ions and protection of proteins from oxidative modifications that introduce carbonyl groups. Conditions that inhibited Mn accumulation or Mn redox cycling rendered D. radiodurans radiation sensitive and highly susceptible to protein oxidation. X-ray fluorescence microprobe analysis showed that Mn is globally distributed in *D. radiodurans,* but Fe is sequestered in a region between dividing cells. For a group of phylogenetically diverse IR-resistant and IR-sensitive wild-type bacteria, our findings support the idea that the degree of resistance is determined by the level of oxidative protein damage caused during irradiation. We present the case that protein, rather than DNA, is the principal target of the biological action of IR in sensitive bacteria, and extreme resistance in Mn-accumulating bacteria is based on protein protection.

## Author Summary

One original goal of radiobiology was to explain why cells are so sensitive to ionizing radiation (IR). Early studies in bacteria incriminated DNA as the principal radiosensitive target, an assertion that remains central to modern radiation toxicity models. More recently, the emphasis has shifted to understanding why bacteria such as Deinococcus radiodurans are extremely resistant to IR, by focusing on DNA repair systems expressed during recovery from high doses of IR. Unfortunately, as key features of DNA-centric hypotheses of extreme resistance have grown weaker, the study of alternative cellular targets has lagged far behind, mostly because of their relative biological complexity. Recent studies have shown that extreme levels of bacterial IR resistance correlate with high intracellular Mn(II) concentrations, and resistant and sensitive bacteria are equally susceptible to IR-induced DNA damage. The current work establishes a mechanistic link between Mn(II) and protection of proteins from radiation damage. In contrast to resistant bacteria, naturally sensitive bacteria are highly susceptible to IR-induced protein oxidation. We propose that sensitive bacteria sustain lethal levels of protein damage at radiation doses that elicit relatively little DNA damage, and that extreme resistance in bacteria is dependent on protein protection.

## Abstract 3

A high intracellular concentration of manganese in*Deinococcus radiodurans* protects proteins, but not DNA, from ionizing radiation-induced oxidative damage. Protein protection may be critical to the known radiation resistance of these bacteria.

## Introduction

The amount of DNA damage caused by a given dose of γ-radiation for resistant and sensitive bacteria is very similar [1,2]. Yet, the range of ionizing radiation (IR) resistances is large [1–3], with a factor of 200 separating the most-resistant from the most-sensitive species [1]. For example, Deinococcus radiodurans can survive levels of IR (10 kGy) that induce approximately 100 DNA double-strand breaks (DSBs) per genome, whereas *Shewanella oneidensis* is killed by levels of IR (0.07 kGy) that result in less than 1 DSB per genome [1]. We have reported a relationship between intracellular Mn/Fe concentration ratios and bacterial survival following exposure to IR, in which the most-resistant cells contained about 300 times more Mn and about three times less Fe than the most-sensitive cells [1]. Furthermore, restricting Mn(II) during growth of D. radiodurans significantly lowered the Mn content of wild-type cells, and IR resistance to levels quantitatively similar to several highly sensitive D. radiodurans DNA repair mutants [1]. However, the nature of Mn-facilitated IR resistance was undefined, and the question of why many bacteria that encode a complement of repair functions are killed by doses of IR that cause little DNA damage has not been resolved [1,4,5].

Broad-based bioinformatic and experimental studies have converged on the conclusion that D. radiodurans uses a relatively conventional set of DNA repair and protection functions, but with far greater efficiency than IR-sensitive bacteria [1–12]. Despite these efforts, however, the molecular mechanisms underlying the extraordinary IR resistance of D. radiodurans and other Mn-accumulating bacteria remain poorly understood [3,4]. For example, recent work by Zahradka et al (2006) [7] showed that DNA polymerase I (PolA) of D. radiodurans supports very efficient DNA replication at the earliest stages of recovery, and could account for the high fidelity of RecA-dependent DSB fragment assembly [7]. However, IR-sensitive *D. radiodurans polA* mutants are fully complemented by expression of the *polA* gene from the IR-sensitive Escherichia coli [12].

The reason why repair proteins, either native or cloned, in D. radiodurans cells function so much better after irradiation than in other organisms is unknown. We show that the amount of protein damage caused by a given dose of γ-radiation for intrinsically resistant and sensitive bacteria is very different. High levels of protein protection during irradiation correlated with high intracellular Mn/Fe concentration ratios and high levels of resistance, whereas proteins in radiation-sensitive cells were highly susceptible to IR-induced oxidation.

## Results

### Mn(II) Ions Protect Protein, but Not DNA, during In Vitro Irradiation

**Figure 1:**
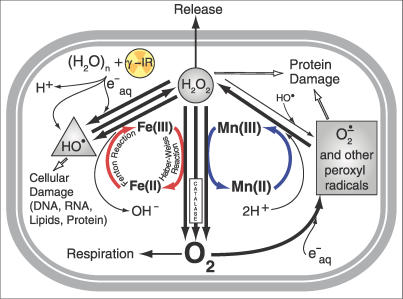
Model of IR-Driven Mn and Fe Redox Cycling (H_2_O)_n_ + γ-IR, radiolysis of water exposed to ionizing radiation [15]: H_2_O→ HO^•^ + H^+^ + e^−^ (IR-induced solvated electron); Primary radiolytic reaction [15]: 2 HO^•^→ H_2_O_2_; IR-induced superoxide [15]: O_2_ + e^−^ → O_2_^•−^; Fenton reaction [15]: Fe(II) + H_2_O_2_ → Fe(III) + HO^•^ + OH^−^ (hydroxide ion); Haber-Weiss reaction [15]: Fe(III) + HO_2_^•^ → Fe(II) + O_2_ + H^+^ and 2 Fe(III) + H_2_O_2_ → 2 Fe(II) + O_2_ + 2 H^+^; Mn oxidation [13]: Mn(II) + O_2_^•−^ + 2 H^+^→ Mn(III) + H_2_O_2_; Mn reduction [13]: 2 Mn(III) + H_2_O_2_ → 2 Mn(II) + O_2_ + 2 H^+^. Under IR, Fe(II,III) redox cycling is predicted to generate HO^•^ and O_2_^•−^, whereas Mn(II,III) redox cycling is predicted to favor O_2_^•−^ scavenging without HO^•^ production.

In comparison to Fe(II), Mn(II) does not significantly react with dioxygen (O_2_) or hydrogen peroxide (H_2_O_2_) at physiological pH values in water. However, Mn(II) has been reported to react strongly with superoxide radicals (O_2_^•−^). For example, as free ions or when complexed with lactate or succinate, Mn(II) can act as a potent scavenger of O_2_^•−^, with Mn cycling between the divalent and trivalent states, releasing H_2_O_2_ as an intermediate [13]. In contrast, when complexed with bicarbonate, Mn(II) catalyzes the disproportionation of H_2_O_2_ [14]. Thus, the presence of Mn might affect the relative abundance of reactive oxygen species (ROS) generated during irradiation. During the γ-radiolysis of water, solvated electrons (e^−^_aq_) react rapidly with O_2_ to form O_2_^•−^ [15], which could react with Mn(II) and protons (H^+^) to form H_2_O_2_ and Mn(III) [13]. Mn redox cycling would occur if Mn(III) generated during irradiation was reduced back to Mn(II) by an electron donor such as H_2_O_2_, a major and relatively stable product of the radiolysis of water (Figure 1).

The primary oxygen radicals generated in the radiolysis of water are hydroxyl radicals (HO^•^) and peroxyl radicals (R-O_2_^•^) [15]. On the basis of the different reactivities of HO^•^ and O_2_^•−^ with DNA and proteins, we tested the ability of Mn(II) to scavenge these ROS during irradiation. DNA is readily damaged by HO^•^, but insensitive to O_2_^•−^ [16]. In contrast, O_2_^•−^ damages [4Fe-4S] cluster-containing enzymes such as aconitase [16], and Mn(II) has been reported to protect enzymes from Fe-catalyzed inactivation by O_2_ in the presence of electron donor systems [17,18]. For example, the restriction enzyme BamHI is readily deactivated by ROS generated under aerobic conditions in the presence of Fe(II) and ascorbate, but not when O_2_ is absent or 4 mM Mn(II) is present [19].

**Figure 2:**
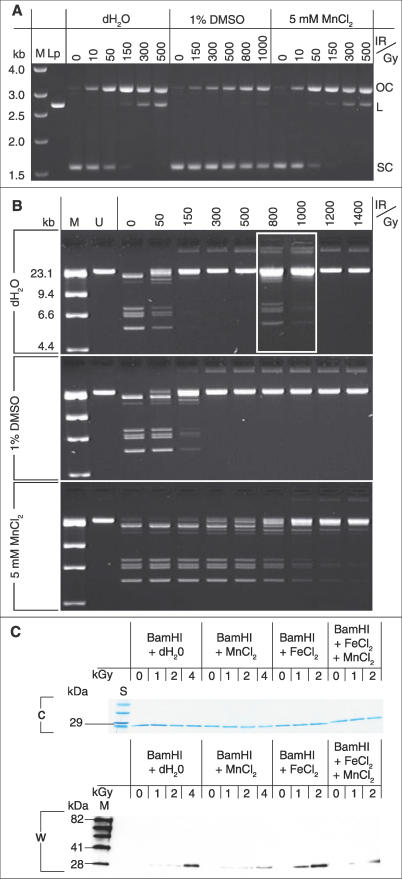
Mn(II) Protects Proteins, but Not DNA, during In Vitro Irradiation (A) DMSO-mediated DNA protection. pUC19 plasmid DNA was irradiated aerobically to the indicated doses in dH_2_O, 1% DMSO (HO^•^ scavenger), or 5 mM MnCl_2_, followed by agarose gel electrophoresis (AGE). MnCl_2_ and DMSO were prepared in dH_2_O. IR, ^60^Co, aerobic at 0 °C. L, linear (2,686 base pairs); Lp, pUC19 + BamHI; M, size markers; OC, open circular; SC, supercoiled. (B) Mn(II)-mediated protein protection. BamHI enzyme was irradiated aerobically to the indicated doses in dH_2_O, 1% DMSO, or 5 mM MnCl_2_, and then incubated with λ-phage DNA for 1 h at 37 °C, followed by AGE. Inset (white border), top gel (dH_2_O): BamHI irradiated anaerobically. M, size markers; U, uncut λ-DNA. (C) Western blot immunoassay of protein-bound carbonyl groups in BamHI irradiated aerobically to the indicated doses in the presence or absence of 5 mM MnCl_2_ and/or 200 μM FeCl_2_. Approximately 220-ng BamHI were loaded per lane in the Western blot (W) and in the Coomassie-stained polyacrylamide denaturing gel (C); M, mixture of artificial IgG-binding protein standards; S, wide-range protein standards.

Consistent with the propensity of HO^•^, but not O_2_^•−^, to damage DNA dissolved in double-distilled de-ionized water (dH_2_O) [15,16], 1% dimethylsulphoxide (DMSO, a HO^•^ scavenger) [15] conferred substantial protection on supercoiled plasmid DNA in vitro during aerobic irradiation, but 5 mM Mn(II) did not (Figure 2A). We also tested BamHI for its susceptibility to IR damage in vitro (Figure 2B and 2C). The highest IR dose that BamHI could survive and then function after irradiation under aerobic conditions in dH_2_O was approximately 50 Gy; in 1% DMSO, it was approximately 150 Gy; and in 5 mM MnCl_2_, it was approximately 1,000 Gy. Since the deactivating IR dose for BamHI that has been irradiated anaerobically in dH_2_O in the absence of DMSO or 5 mM MnCl_2_ was approximately 1,000 Gy (Figure 2B), we examined whether free Mn(II) ions might protect BamHI from O_2_-dependent modifications during irradiation. In vitro, 5 mM MnCl_2_ limited IR-induced oxidative protein damage under aerobic conditions in the presence or absence of Fe, as measured by assaying for carbonyl group (aldehydes and ketones) generation into proteins at Lys, Arg, Pro, and Thr residues by oxidative reactions [20] (Figure 2C). The level of carbonyl groups in proteins is widely used as a marker of oxidative protein damage and has attracted a great deal of attention due to its irreversible and unrepairable nature [21]. Since not all oxidative modifications lead to carbonyl derivatives, however, the levels of oxidation detected represent minimal values. These results support our model that Mn(II) ions might protect proteins by scavenging peroxyl radicals (O_2_^•−^, HO_2_^•^, and R-O_2_^•^) and/or H_2_O_2_, but do not scavenge HO^•^ generated during irradiation (Figure 1).

### Bacterial Radioresistance Is Quantifiably Related to Protein Oxidation

**Figure 3:**
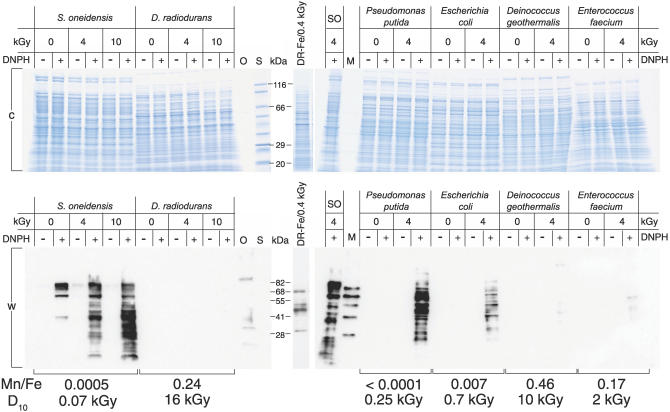
In Vivo IR-Induced Oxidative Protein Damage Samples were treated (+) or not treated (−) with DNPH. For the Western blot (W) and for the Coomassie-stained polyacrylamide denaturing gel (C), 20-μg protein samples were loaded per lane. DR/Fe/0.4 kGy indicates that the D. radiodurans (non-irradiated) cell extract was adjusted to 500 μM FeCl_2_ and exposed to 0.4 kGy in vitro. Values for intracellular Mn/Fe concentration ratios and D_10_ at the bottom of the figure, as reported previously [1]. M, mixture of artificial IgG-binding protein standards; O, oxidized protein standards; S, wide-range protein standards; SO, S. oneidensis.

**Figure 4:**
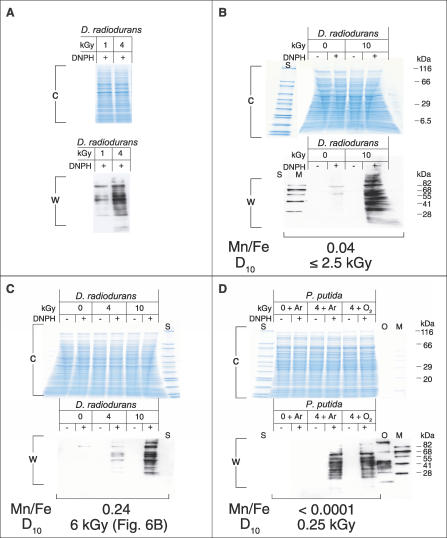
Additional Oxidative Protein Damage Assays (A) In vitro IR-induced oxidative protein damage. Western blot (W) immunoassay of protein-bound carbonyl groups in D. radiodurans (non-irradiated) cell extract adjusted to 500 μM FeCl_2_ and irradiated to the indicated doses. A total of 20 μg of protein extract loaded per lane. (B) Mn-depleted, radiosensitive D. radiodurans cells [1] are highly susceptible to oxidative protein damage during irradiation. D. radiodurans was grown in defined rich medium without Mn supplementation (no-Mn DRM) [1] to OD_600_ 0.8 and exposed aerobically to 10 kGy. A total of 20 μg of protein extract loaded per lane. (C) Decreased survival of D. radiodurans irradiated at pH 10.5 correlates with oxidative protein damage. D. radiodurans was grown to OD_600_ approximately 0.9 in TGY (pH 7), adjusted to pH 10.5, and exposed aerobically to the indicated doses. A total of 20 μg of protein extract loaded per lane. (D) *P. putida* proteins are similarly susceptible to oxidative protein damage when cells are irradiated anaerobically (+Ar) or aerobically (+O_2_). P. putida was grown to OD_600_ approximately 0.9 in TGY, purged with ultra-high purity Ar, and irradiated in sealed tubes to 4 kGy. Values for intracellular Mn/Fe concentration ratios and D_10_ at the bottom of (B), (C), and (D), as reported previously [1]. A total of 20 μg of protein extract loaded per lane. C, Coomassie-stained polyacrylamide denaturing gel; M, mixture of artificial IgG-binding protein standards; O, oxidized protein standards; S, wide-range protein standards; +, DNPH treated;−, DNPH untreated.

To demonstrate a mechanistic link between solution-phase radiochemistry of Mn ions (Figure 2) and their physiological targets in vivo, we examined IR-induced protein damage in IR-sensitive and IR-resistant bacteria (Figure 3). Cellular proteins in the most-sensitive bacteria were substantially more vulnerable to IR-induced oxidation than proteins in the most-resistant bacteria (Figure 3); and from the pattern of oxidized bands, we infer that not all proteins in sensitive bacteria are equally susceptible to carbonylation. At 4 kGy, high levels of protein oxidation occurred in cells with the lowest intracellular Mn/Fe concentration ratios, whereas no protein oxidation was detected in cells with the highest Mn/Fe ratios (see bottom of Figure 3 for bacterial IR survival values and Mn/Fe concentration ratios). In vitro, proteins from resistant bacteria were readily carbonylated when exposed to IR in the presence of Fe (Figure 4A), which confirmed that proteins in resistant bacteria are not inherently resistant to oxidation. Furthermore, we previously reported that D. radiodurans cells grown in defined rich medium without Mn supplementation (No-Mn DRM) were depleted in Mn and, at 10 kGy, displayed a 1,000-fold reduction in survival compared to cells with normal Mn concentrations [1]. D. radiodurans cells grown in DRM without Mn were highly susceptible to protein oxidation during irradiation (Figure 4B). In comparison, D. radiodurans cells with normal intracellular Mn concentrations were sensitized to IR and protein oxidation when irradiated at pH 10.5 (Figure 4C). Pseudomonas putida cells irradiated anaerobically were equally sensitive to IR and as susceptible to IR-induced protein carbonylation as cells irradiated aerobically (Figure 4D). Thus, high levels of IR-induced protein oxidation in bacteria correlated with IR sensitivity in the presence or absence of atmospheric O_2_, and the IR resistance and level of protein oxidation in D. radiodurans cells with normal intracellular Mn concentrations could be controlled exogenously.

### Generation of O_2_ and H_2_O_2_ in Anaerobic MnCl_2_ Solutions during Irradiation

**Figure 5:**
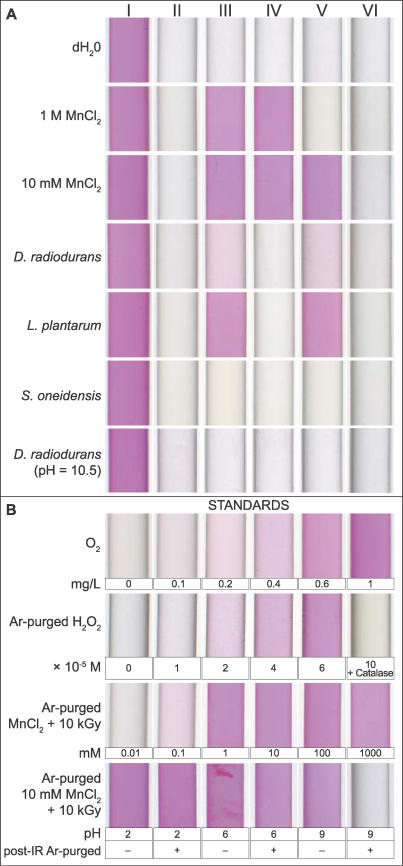
Mn-Dependent IR-Driven O_2_ and H_2_O_2_ Generation (A) Column I. No IR, aerobic; II. No IR, anaerobic (pre-conditioned by purging with Ar [ultra-high purity]); III. 10 kGy, anaerobic; IV. 10 kGy, anaerobic, followed by incubation at 32 °C for 60 min; V. As for column III, but re-purged with Ar after irradiation; and VI. As for column III, but treated with catalase (15,000 units) after irradiation and then re-purged with Ar. O_2_/H_2_O_2_ concentration determined by the Rhodazine D assay. See Figure S2 for additional assays. Irradiations (^60^Co) were at 0 °C. MnCl_2_ solutions and bacteria (1.6 × 10^9^ cells/ml) were prepared in dH_2_O (pH ∼6). (B) Standards.

In vitro, the stoichiometry of intermediates and end products of Mn redox cycling is dependent on the concentration of reactants [13]. For example, Mn(III) accumulates if H_2_O_2_ becomes limiting, whereas an excess of O_2_^•−^ and H^+^ favors the formation of H_2_O_2_ [13] (Figure 1). Using assays based on Rhodazine D, a sensitive colorimetric reagent for measuring O_2_ and H_2_O_2_, we tested whether or not MnCl_2_ solutions exposed anaerobically to 10 kGy generated these species (Figures 5A and S1). Dissolved O_2_ and H_2_O_2_ react with the pale yellow leuco form of Rhodazine D to produce a rose color, with the color proportional to the dissolved O_2_ or H_2_O_2_ concentration. Color development in an Ar-purged solution tested after irradiation (Figure 5A, column III), but not upon re-purging with Ar (Figure 5A, column V), indicated O_2_ formation. Color development in an Ar-purged solution tested after irradiation and upon re-purging with Ar (Figure 5A, column V), but not following catalase treatment (Figure 5A, column VI), indicated H_2_O_2_ accumulation. Consistent with the existence of a threshold concentration of Mn(II) needed for Mn redox cycling [13] under in vitro irradiation were (1) exposure of anaerobic dH_2_O or MnCl_2_ solutions at concentrations below 0.1 mM to 10 kGy did not generate detectable levels of O_2_ or H_2_O_2_ (Figure 5A and 5B); (2) exposure of anaerobic MnCl_2_ solutions at intermediate concentrations (0.1–10 mM) to 10 kGy generated H_2_O_2_ (Figure 5A); and (3) from initially anaerobic conditions, exposure of MnCl_2_ solutions at high concentrations (1 M) to 10 kGy yielded O_2_, but not H_2_O_2_ (Figure 5A), and copious Mn dioxide precipitates (Figure S2); we infer that the accumulation of Mn dioxides was caused by a shortage of H_2_O_2_ [13], which is decomposed by γ-radiolysis [15].

### Mn-Accumulating Cells Release H_2_O_2_ during Irradiation

**Figure 6:**
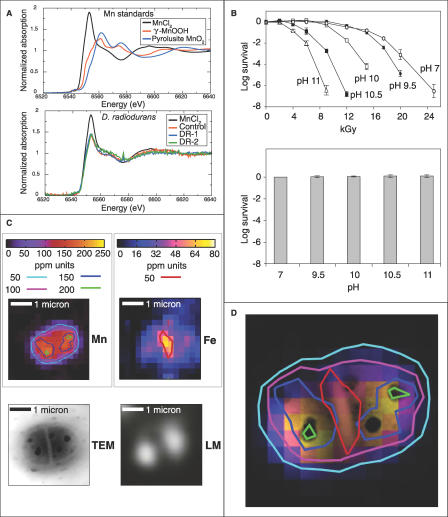
IR Resistance and Mn Profiles of D. radiodurans (A) XANES absorption spectra. Top, Mn standards: MnCl_2_ (Mn(II)), γ-MnOOH (Mn(III)), and MnO_2_ (Mn(IV)). Bottom, D. radiodurans. Control: No IR, analyzed frozen (−14 °C). DR-1: +10 kGy (−78 °C), analyzed frozen (−14 °C). DR-2: +10 kGy (−78 °C), analyzed thawed (5 °C). (B) pH-dependent IR survival of D. radiodurans. Top, cells were grown in TGY (pH 7), irradiated (^60^Co) in TGY (0 °C) at the indicated pH, neutralized, and then recovered on TGY (pH 7). Bottom, survival of non-irradiated D. radiodurans grown in TGY (pH 7), then held in TGY (0 °C) at the indicated pH for 16 h, neutralized, and then plated on TGY (pH 7). IR survival assays as described previously [1]. (C) XRF elemental distribution maps of Mn and Fe in D. radiodurans. The D. radiodurans diplococcus (designation: no. 109) was isolated from the mid-logarithmic growth phase (OD_600_ 0.3). For additional XRF microprobe analyses (P, Cl, Mn, Fe, Co, Ni, and Cr), see Figure S3. (D) Transparent image overlay of TEM, LM, and XRF measurements, and ppm contour lines displayed in (C).

The obligate aerobic D. radiodurans accumulates 2 mM or greater Mn (1 × 10^5^ Mn atoms/cell, assuming an average cell volume of 6.5 × 10^−2^ μm^3^) [1], which is present in cells as Mn(II) (Figure 6A) [1], and the facultative anaerobic, radioresistant bacterium Lactobacillus plantarum [1,22] accumulates 20–25 mM Mn(II) [23]. For an irradiated cell containing 2–25 mM Mn engaged in catalytic Mn redox cycling [13], intracellular O_2_^•−^, which does not easily cross biological membranes [16,24], might be reduced to H_2_O_2_, which is membrane permeable and could diffuse out of the cell and accumulate (Figure 1). When exposed to 10 kGy as cell suspensions in dH_2_O at 0 °C and pre-conditioned to be anaerobic, H_2_O_2_ was released by D. radiodurans (∼2 × 10^−5^ M) and L. plantarum (∼6 × 10^−5^ M), in which the presence of H_2_O_2_ after irradiation was confirmed by catalase-treatment (Figure 5A); notably, H_2_O_2_ was consumed by the cells upon incubation at 32 °C (Figure 5A). We infer that H_2_O_2_ was produced by intracellular Mn(II,III) redox cycling. In contrast, detectable levels of H_2_O_2_ or O_2_ were not released by non-irradiated D. radiodurans or *L. plantarum* control samples, nor by irradiated S. oneidensis (Figure 5A), which accumulates substantially more Fe than Mn and is extremely sensitive to IR [1,5].

### Increasing pH Inhibits Mn Redox Cycling

Reducing the equilibrium concentration of H^+^ with hydroxide ions (OH^−^) is predicted to limit Mn redox cycling [13] and the accumulation of H_2_O_2_ (Figure 1). In testing the possibility that survival of irradiated D. radiodurans is vulnerable to conditions that limit intracellular acidification, we first established that IR-driven Mn redox cycling could be inhibited in vitro by increasing the pH. In vitro, exposure to IR of anaerobic 10 mM MnCl_2_ at pH 9 or higher yielded O_2_ but not H_2_O_2_, whereas H_2_O_2_ was formed at pH values below 9 (Figure 5B). Consistent with the prediction that Mn redox cycling can be inhibited in vivo by OH^−^, D. radiodurans irradiated at pH 10.5 did not release H_2_O_2_ or O_2_ (Figure 5A). In vivo, the 10% survival value (D_10_) [1] of D. radiodurans cells grown, irradiated, and recovered at pH 7 was 16 kGy, whereas the D_10_ was 6 kGy for cells grown at pH 7, irradiated at pH 10.5, and recovered at pH 7 (Figure 6B, top); survival of non-irradiated D. radiodurans control samples held at a pH of 11 or lower for 16 h was approximately 100% (Figure 6B, bottom). In vivo, the pH-dependent loss in IR resistance of D. radiodurans (Figure 6B, top) correlated with a substantial increase in oxidative protein damage during irradiation (Figure 4C). Thus, at pH values at which IR-driven Mn redox cycling was inhibited in vitro (Figure 5B), the IR resistance of D. radiodurans was significantly decreased, and the cells were highly susceptible to IR-induced protein oxidation (Figure 4C).

### Distribution of Mn and Fe in D. radiodurans

Because the formation of ROS during irradiation is extremely rapid [15], an intracellular protection system which is ubiquitous, but not highly dependent on the induction of enzymes, stage of growth, or temperature over a range at which cells are metabolically active, could provide a selective advantage to the host in some environments. In this context, we examined the intracellular distribution of Mn and Fe in D. radiodurans cells using X-ray fluorescence (XRF) microprobe analysis [25] (Figures 6C and S3). Within a representative diplococcus, whereas Mn(II) was globally distributed, Fe was partitioned largely outside the cytoplasm in a region overlapping the septum between dividing cells (Figure 6D).

## Discussion

We previously demonstrated a critical role for the accumulation of Mn(II) in D. radiodurans in a mechanism toward surviving IR that is not dependent on Mn-SOD (Mn-dependent superoxide dismutase) [1]. D. radiodurans contains four to ten identical copies of its genome per cell, and when irradiated to a dose of 10 kGy, generates more than 400 genomic DSB fragments per cell [1,8,26]. Yet, this amount of DNA damage in D. radiodurans does not typically lead to cell death [1,8]. Bioinformatic and experimental reports generally support that genome configuration and copy number, and enzymatic protection and repair functions of D. radiodurans do not have unique properties that are essential or prerequisite for expression of the extreme-resistance phenotype [1–12]. For example, D. radiodurans DNA repair and protection genes do not differ greatly from their counterparts in the IR-sensitive *S. oneidensis, P. putida,* or E. coli [1,6]; several E. coli DNA repair genes have been shown to fully restore corresponding radiation-sensitive D. radiodurans mutants to wild-type levels of D. radiodurans resistance [12,27]; Mn-SOD is not needed for survival of D. radiodurans following acute irradiation or growth under 50 Gy/h [1,11]; non-homologous end joining of D. radiodurans chromosomal DSB fragments following IR is not observed [28]; and the products of interchromosomal recombination in D. radiodurans following irradiation are consistent with the canonical version of the DSB repair model [8]. Over the last decade, several hypotheses to reconcile these findings have built on the idea that D. radiodurans might use mechanisms that restrict the diffusion of DNA DSB fragments produced following irradiation, to facilitate repair [3,29]. However, transmission electron microscopy [TEM] of Mn-depleted, radiosensitive D. radiodurans displayed normal levels of chromosomal condensation [1], and cryoelectron microscopy of vitreous sections of D. radiodurans supports the conclusion that DNA fragments in D. radiodurans are mobile and that the arrangement of its nucleoids does not play a key role in radioresistance [10,30]. Consistently, a series of earlier molecular studies on irradiated D. radiodurans cells showed high levels of recombination between homologous DSB fragments originating from widely separated genomic locations [8,28,31]. Evidence presented here supports the idea that the extreme-resistance phenotype of D. radiodurans and other bacteria with high intracellular Mn/Fe concentration ratios is dependent on a mechanism that protects proteins from oxidative damage during irradiation, which could offset the need for highly specialized cellular repair systems.

Previous work has shown that the linear density of DSBs introduced into genomic DNA by a given dose of IR in extremely resistant and sensitive bacteria is essentially the same [1,2]. In contrast, we find that protein damage is quantifiably related to bacterial radioresistance (Figures 3, 4B, and 4C). The most-sensitive cells had very low Mn/Fe concentration ratios and were highly susceptible to IR-induced protein oxidation, whereas the most-resistant cells had high intracellular Mn/Fe ratios and were relatively insusceptible to protein oxidation. Although the mechanism by which Mn protects proteins during irradiation remains unknown, our results provide insight into how Mn redox cycling could attenuate the detrimental effects of Fe redox cycling.

Scavenging of ROS in IR-resistant bacteria may be linked to both the presence of Mn and relatively low cytosolic levels of Fe (Figure 6C and 6D) [1]. Our observation that resistant bacteria released H_2_O_2_ during irradiation, but sensitive bacteria did not (Figure 5A), is consistent with the idea that Fe redox cycling is limited in Mn-accumulating cells. Most bacteria accumulate near-millimolar concentrations of intracellular Fe, primarily for assembly of Fe-S and haem proteins [1,32,33]. However, resistant bacteria typically encode fewer proteins with Fe-S domains than sensitive bacteria [4]. Since IR-induced ROS likely damage exposed Fe-S clusters, releasing Fe(II) [16,32], Fe-laden sensitive cells might be predisposed to Fe redox cycling reactions during irradiation. Since 5 mM Mn(II) does not significantly scavenge HO^•^ (Figure 2A), Mn redox cycling likely does not protect cells from HO^•^ generated either directly by water radiolysis or indirectly by the Fenton (Fe(II)) reaction (Figure 1) [15]. However, the Haber-Weiss (Fe(III)) reaction (Figure 1) [15] generates O_2_, which under IR would give rise to O_2_^•−^ and other peroxyl radicals (R-O_2_^•^) (Figure 1) [15]. Compared to HO^•^, O_2_^•−^ is relatively unreactive with a large number of compounds including DNA, but can undergo chain reactions leading to organic hydroperoxides [15], which decompose in the presence of Fe to give new radicals, including oxidizing alkoxyl radicals that are more reactive than peroxyl radicals [15,34]. Although intracellular Mn(II) does not protect cells from DNA damage caused by HO^•^ [1], scavenging of simple peroxyl radicals by Mn redox cycling might prevent the propagation of secondary reactions that ultimately damage proteins [15,16,34].

The exceptionally high catalase activities of D. radiodurans [11] might be expected to favor the accumulation of Mn(III) (Figure 1), a strong oxidant capable of damaging cell components [13]. We did not detect significant levels of Mn(III) or Mn(IV) in irradiated or non-irradiated D. radiodurans by X-ray-absorption near-edge structure (XANES) spectroscopy (Figure 6A), indicating that Mn(III) might also be reduced by other mechanisms in vivo. Notably, when Mn is complexed with succinate or lactate in vitro, the efficiency of Mn redox cycling is greatly increased, such that Mn(III) reactivity is similar to that of Mn(II) complexes and re-reduction of Mn(III) by O_2_^•−^ might occur [13]. In this context, metabolic pathway switching in D. radiodurans cells immediately after irradiation has been reported to favor the production of succinate via up-regulation of the glyoxylate bypass of its tricarboxylic acid (TCA) cycle, and down-regulation of degradative steps of the TCA cycle [4,5,9]. In bicarbonate/CO_2_ buffer, Mn(II) is reported to catalyze the oxidation of free amino acids such as leucine and alanine by H_2_O_2_ and the dismutation of H_2_O_2_ [35]. Thus, complexes containing Mn and amino acids or organic acids might scavenge H_2_O_2_ in addition to O_2_^•−^ in cells exposed to IR.

An interesting feature of the systems for energy production in D. radiodurans is that, unlike most other free-living bacteria, it uses the vacuolar type of proton ATP synthase instead of the F_1_F_0_ type [6]. Vacuolar (V)-type H^+^-ATPases are typical of eukaryotes and archaea, and central players in intracellular acidification [36], which might facilitate Mn redox cycling by providing H^+^ (Figure 1). In this context, our findings generally support bioinformatic studies by Karlin and Mrazek in 2001 [37], who proffered a new explanation for the resistance of D. radiodurans contingent on a role of predicted highly expressed (PHX) genes for proteases, the glyoxylate bypass of the TCA cycle, ABC-type transporters of amino acids and Mn, and (V)-type ATPases.

The speciation, distribution, and relatively high concentration of Mn in D. radiodurans [1] (Figure 6A and 6C) support the idea that Mn(II) could provide immediate cytosolic protection from O_2_^•−^, and facilitate removal of H_2_O_2_ from cells exposed to IR. Further, electron-dense granules (EDGs) (Figure 6C, TEM, circular dark ∼0.2 μM inclusions), which are frequently observed in electron microscopy images at the center of D. radiodurans nucleoids [1,4,10], were associated with the highest regional Mn concentrations (200 parts per million [ppm]; 3.6 mM) (Figure 6D), perhaps to protect enzymic DNA repair functions, many of which are dependent on redox-active [4Fe-4S] clusters [38]. Importantly, our findings do not preclude the possibility that intracellular Mn(II) also prevents lipid peroxidation in cell membranes. In this context, however, the lowest regional Mn concentrations (50 ppm) were associated with the cell envelope [6] (Figures 6C and S3), indicating that Mn(II) predominantly protects the cytosol; and earlier reports strongly support the idea that lipid peroxidation can be dissociated from lethal damage in irradiated mammalian and irradiated bacterial cells [34,39]. During recovery of irradiated *D. radiodurans,* additional damage might be limited by secondary antioxidant defenses, including attendant cellular responses that limit the production of metabolism-induced ROS [4,9], and degradation of oxidized proteins by the expanded family of subtilisin-like proteases [6]. The Mn content of bacteria [1] might also determine the amount of protein damage caused in cells exposed to other oxidative stress-inducing conditions, including desiccation [1,6,24] and ultraviolet (UV) radiation [5,12], and xenobiotic agents such as Cr(VI) and mitomycin-C (MMC) that elicit redox-related toxicity [40].

Chromosomal DNA is an indispensable molecule whose integrity must be conserved following exposure of a cell to IR to ensure survival [15], such that the functionality of DNA repair and replication systems ultimately determines if an irradiated cell lives or dies, even for the most IR-resistant bacteria [6,12,27]. Our findings that IR-induced cellular protein damage (Figures 3 and 4), but not DNA damage [1,2], is quantifiably related to radioresistance, and intracellular Mn/Fe concentration ratios could help explain why bacteria that encode a similar repertoire of DNA repair functions display such large differences in IR resistance [1,3,5]. Specifically, we propose that redox cycling of Mn(II) that is accumulated in resistant bacteria [1] protects proteins from oxidation during irradiation (Figure 1), with the result that enzyme systems involved in recovery survive and function with great efficiency. This could explain why the *polA* gene of E. coli fully complements IR-, UV- and MMC-sensitive *D. radiodurans polA* mutants [12]. In comparison, we attribute the high level of radiation sensitivity of Fe-rich, Mn-poor bacteria to their susceptibility to global Fe-mediated oxidative protein damage during irradiation under aerobic or anaerobic conditions (Figure 4D). Oxidative modification of proteins by IR could disrupt cellular functions involved in DNA repair either by loss of catalytic and structural integrity or by interruption of regulatory pathways, which in extremely radiation-sensitive cells might render protein damage lethal before significant DNA damage has accumulated [5]. At sublethal IR doses in sensitive cells, oxidatively damaged DNA repair enzymes would be expected to passively promote mutations by misrepair. Oxidized proteins, however, might also actively promote mutation by transmitting damage to other cellular constituents, including DNA [34,41]. In conclusion, our data provide a novel framework for understanding how intracellular Mn and Fe contribute to IR resistance, which is important since these findings may come to affect models of radiation toxicity, as well as approaches to control recovery from radiation injury [42], including the development of systems for delivery into cells of Mn-based radioprotective complexes or Fe-based radiosensitizers [43].

## Materials and Methods

### Strains.

The wild-type strains used were as follows: D. radiodurans (ATCC BAA-816); D. geothermalis (DSM 11300); S. oneidensis (MR-1) (ATCC 700550); P. putida (ATCC 47054); Enterococcus faecium (ATCC 19434); L. plantarum (ATCC 14917); and E. coli (K-12) (MG1655).

### Growth and γ-irradiations.

Strains were cultured aerobically in undefined liquid rich medium [1] (TGY: 1% Bacto-tryptone, 0.1% glucose, 0.5% yeast extract) (pH 7) at 32 °C to an optical density at 600 nm (OD_600_) of 0.9–1.0, unless indicated otherwise. For anaerobic irradiations, 6-ml 0–1,000 mM MnCl_2_ solutions in dH_2_O (pH ∼6), or approximately 1 × 10^10^ bacterial cells (∼5 × 10^9^ diplococci) re-suspended in 6-ml dH_2_O, were transferred to Quick-Seal ultracentrifuge tubes (13 cm^3^) (Beckman Instruments, Palo Alto, California, United States), unless indicated otherwise. The tubes were purged at room temperature with ultra-high purity Ar (99.999%) (ValleyNational Gases, Frederick, Maryland, United States) for 5 min (200 cm^3^/min), sealed anaerobically, and irradiated on ice (0 °C) at 1.8 Gy/s (^60^Co, Model 109; J. L. Shepard and Associates, San Fernando, California, United States).

### pUC19 and BamHI IR sensitivities.

The pUC19 assay was performed as follows. Supercoiled pUC19 (1 mg/ml) (New England Biolabs, Ipswich, Massachusetts, United States) was diluted (1/25) in dH_2_O, 1% DMSO, or 5 mM MnCl_2;_ 50-μl aliquots of each of the three pUC19 dilutions were irradiated (^60^Co) at 0 °C to the indicated doses. A total of 88 ng of each IR-treated pUC19 sample was subjected to agarose (0.9%) gel electrophoresis in 1 × TBE (Tris, borate, EDTA buffer) and 250-ng/ml ethidium bromide, at 47 V for 14 h. Linearized plasmid (Lp) (pUC19 + BamHI) was used as a marker in gels containing IR-treated supercoiled pUC19.

The BamHI assay was performed as follows. BamHI (700,000 U/ml) (New England Biolabs) was diluted (1/1,500) in dH_2_O, 1% DMSO, or 5 mM MnCl_2_; 1,000-μl aliquots of each of the three BamHI dilutions were irradiated (^60^Co) aerobically at 0 °C to the indicated doses. For anaerobic BamHI irradiations, 5-ml aliquots of diluted BamHI/dH_2_O were transferred to separate Quick-Seal ultracentrifuge tubes (13 cm^3^) (Beckman Instruments), purged at room temperature with ultra-high purity Ar (99.999%) (Valley National Gases) for 5 min (200 cm^3^/min), and sealed anaerobically before irradiation. A total of 40 μl (23 units) of each IR-treated BamHI sample was transferred to separate reaction mixes (final volume, 60 μl) containing 250-ng λ-phage DNA (New England Biolabs), 50 mM NaCl, 10 mM Tris-HCl (pH 7.9), 10 mM MgCl_2_, and 1 mM dithiothreitol. BamHI/λ DNA reactions were incubated for 1 h at 37 °C, followed by agarose (0.7%/1 × TBE) gel electrophoresis at 23 V for 18 h.

### Protein extractions.

The 650-ml cultures of the indicated bacteria grown in TGY to OD_600_ 0.9 were harvested by centrifugation, resuspended in 30 ml TGY, and exposed to IR (0 °C). Irradiated and non-irradiated (control) cells were washed and then resuspended in lysis buffer (50 mM potassium phosphate buffer [pH 7.0], 0 °C). A cell suspension (∼4 × 10^9^ cells/ml, 2–4 ml) was adjusted to 1% (v/v) β-mercaptoethanol and passed through a French pressure cell (0 °C) at 20,000 lb/in^2^, and the lysate centrifuged twice at 12,000 × *g* at 4 °C for 30 min. The protein concentration of a supernatant was determined by the Coomassie (Bradford) assay (BioRad, Hercules, California, United States), and the samples were then diluted with lysis buffer/1% β-mercaptoethanol to 20-μg/μl protein, divided into 50-μl aliquots, and stored at −80 °C. The addition of β-mercaptoethanol prior to cell lysis and for storage was recommended in the protocols accompanying the OxyBlot Oxidation Detection Kit (Chemicon International, Temecula, California, United States) (see below) to prevent the oxidation of proteins during and after cell lysis. Assays for oxidative protein damage (Figure 3) were all conducted in parallel within 3 d of protein extraction.

### Assay for oxidative protein damage.

Protein oxidation in freshly prepared extracts was measured using OxyBlot Protein Oxidation Detection Kit (S7150) (Chemicon International), including the indicated molecular-weight markers. The carbonyl groups in the protein side chains were derivatized to 2,4-dinitrophenylhydrazone by reaction with 2,4-dinitrophenylhydrazine (DNPH) for 15 min in 3% (w/v) SDS. Western blotting: the DNP-derivatized protein samples were separated by polyacrylamide denaturing gel electrophoresis (4%–20% gradient gels; BioRad) at 195 V for 50 min followed by transferring proteins to a nitrocellulose membrane for 40 min (BioRad). The membranes were incubated with primary antibody, specific to the DNP moiety of the proteins. This step was followed by incubation with a horseradish peroxidase-antibody conjugate directed against the primary antibody (secondary antibody: goat anti-rabbit IgG). The membranes were then treated with chemiluminescent (SuperSignal) substrate (Pierce Biotechnology, Rockford, Illinois, United States) and imaged by exposure to light sensitive films (BIOMAX Light Film; Kodak, Rochester, New York, United States).

### Rhodazine D O_2_/H_2_O_2_ assay.

O_2_ and H_2_O_2_ concentrations were determined by the Rhodazine D assay (RDA) (CHEMetrics, Calverton, Virginia, United States). All Quick-Seal centrifuge tubes containing cells were centrifuged at 4 °C (2,000 × *g,* 10 min) after irradiation. Supernatants were tested by the RDA. The RDA is suitable for measuring 0.1–1.0 mg/l O_2_, and 1–10 × 10^−5^ M H_2_O_2_. Mn(III,IV) are oxidants that will also cause a positive RDA test result. However, Mn(III,IV) dioxides are insoluble in water (circa-neutral pH) and readily removed from suspension by centrifugation (2,000 × *g,* 5 min) or standing. RDA results indicating 1 mg/l or more O_2_ or 10 × 10^−5^ M or greater H_2_O_2_ were also tested by the Indigo Carmine assay (CHEMetrics), which yields a blue color suitable for measuring higher concentrations of O_2_ and H_2_O_2_. Once a test solution had filled an assay vial, the open end was sealed anaerobically with vacuum grease, and the vial was stored in the dark.

### XANES.

Mn K-edge XANES spectra were measured [44] in transmission on standard compounds (MnCl_2_, γ-MnOOH, and MnO_2_) and in fluorescence on the D. radiodurans samples. The measurements on γ-MnOOH and MnO_2_ were made on fine powders spread evenly onto adhesive tape and folded to make samples of appropriate thickness for the transmission experiment. The 1 M MnCl_2_ solution and D. radiodurans cell suspensions (OD_600_ 0.9) were absorbed on paper and cellulose acetate filters, respectively, frozen, and then stored at −80 °C. For XANES measurements, samples were transferred from dry ice (−78 °C) to a sample stage consisting of an insulated Peltier stack kept under a helium atmosphere. Such samples were maintained at −14 °C during the analysis. The XANES spectra were measured at the MRCAT [45] (Materials Research Collaborative Access Team) sector 10ID beamline at the Advanced Photon Source (APS). The 10ID is an insertion device beamline using APS undulator-A [46]. The undulator was tapered by approximately 2 keV to reduce the variation in incident radiation to less than 15% over the energy range used in these measurements. The incident X-ray beam was the undulator fundamental and was monochromated using the (111) reflection of a liquid-nitrogen–cooled, double-crystal, silicon monochromator. Higher harmonic content of the incident beam was rejected using a polished float glass mirror. Ionization chambers were used to measure the incident and transmitted intensities, and were filled with 10% nitrogen/90% helium and 100% nitrogen, respectively. The fluorescence spectra were measured with an ionization detector in the Stern-Heald geometry [47] filled with Ar gas, and used a chromium oxide filter of three absorption-lengths thickness to reduce the background signal. The incident X-ray beam was 1 mm square. Linearity tests [48] indicated less than 0.04% variation for a 50% decrease in incident X-ray intensity. The XANES data were processed using the Athena program [49]. All data scans were aligned in energy using a reference spectrum derived from metallic manganese. Between five and 20 scans per sample were averaged to improve measurement statistics. The averaged data were normalized [44] by regressing a line to the pre-edge region between approximately 6,400–6,500 eV. This line was subtracted from the data. A quadratic polynomial was regressed to the region from approximately 6,600 eV to the end of the data range. This quadratic polynomial was extrapolated to the edge energy (6,548.5 eV for the samples containing D. radiodurans). The normalized data were divided by the value of this polynomial at the edge energy. Notably, the edge positions of XANES spectra of irradiated or non-irradiated D. radiodurans samples were essentially the same as for aqueous MnCl_2_ solutions. This is consistent with oxygen atoms surrounding Mn(II) in *D. radiodurans,* and with no significant change in Mn valence during irradiation, which would have been observed in cell samples containing 5% or more Mn(III,IV).

### XRF.

XRF microprobe analysis measurements [25] were made at beamline 2ID-D at the APS [50]. The 2ID-D is an undulator beamline with Fresnel zone plates focusing optics that produced a focal spot with a FWHM (full width at half maximum) spatial resolution of approximately 120 nm for these experiments. A silicon (111) double-crystal monochromator was used to shine 10-keV photons on the Fresnel zone plates. The sample was rastered through the focal spot of the X-ray beam. For each pixel, the full XRF spectrum between approximately 2 keV and 10 keV was measured using a silicon drift detector. Thus, the distribution of elements between phosphorus and zinc on the periodic table of elements could be measured with 120-nm resolution throughout a cell and its periphery. XRF microprobe measurements were made on D. radiodurans cells grown to OD_600_ 0.3. The cells were deposited on grids as suspensions in TGY liquid medium, which served to help maintain the structure and viability of the cells as they dried. The complete results for two distinct diplococci (no. 109 and no. 110) are presented in Figure S3. The position coordinates of individual diplococci mounted on formvar-coated gold TEM grids were determined by light microscopy (LM). TEM grids were subsequently placed in the focal plane of the X-ray optic, and the grid markers were used to locate the targeted cells. Data from 2ID-D were initially processed using the program MAPS [51]. The average XRF spectral intensities from 28 adjacent pixels in the upper right-hand corners of XRF images, in areas beyond the boundaries of a cell (Figure S3), were used to determine the background signal for the elements under investigation. The average background spectrum for an element being mapped was subtracted from all other pixels of the image. Next, a mathematical model representing the original morphology of cells was constructed in approximate likeness to diplococci, which resemble adjoined truncated spheres. Within each pixel, the intensity of an element's XRF signal was weighted by the average thickness of the diplococcus subtended by a respective pixel. The contour lines are straight-line segments between points of equal intensity, in which linear interpolation of the actual data was used to determine the locations of the contour values. The contours were generated using the algorithm Gnuplot (http://www.gnuplot.info). In combination, this approach limited the effect of cell thickness on the elemental distribution maps, and facilitated correlation between electron-dense granules (EDGs) and Mn hotspots. Element distributions are presented as mass parts per million (ppm).

### TEM.

Whole cells deposited on formvar-coated gold TEM grids and analyzed by XRF microprobe analysis were subsequently imaged in a FEI Tecnai TF30 transmission electron microscope equipped with a field-emission gun and operated at an accelerating voltage of 300 kV. An objective aperture with a diameter of 40 μm was selected to optimize contrast due to differences in elastic scattering across the specimen. Bright-field electron micrographs were recorded using a Gatan Ultrascan Model 894 cooled CCD camera containing four mega-pixels and operated with a four-port parallel read-out. Images were analyzed using Gatan Digital Micrograph software (version 3.4). Pixel intensities were transformed to a logarithmic scale to visualize the EDGs (∼ 0.2 μm in diameter) in cells.

### LM.

LM was used to identify the position coordinates of discrete diplococci on formvar-coated gold TEM grids before XRF microprobe analysis (Figure S3); gold grids were supported by glass slides during LM. LM images were captured using a Leica DM RXA epi-fluorescent microscope (Leica, Wetzlar, Germany). Differential interference contrast images were recorded using a Scion Corporation (Frederick, Maryland, United States) CCD camera and analyzed with xyzGrabber.

## Supporting Information

Figure S1 Additional Assays for O_2_/H_2_O_2_
(A) Indigo carmine assay (ICA) (CHEMetrics). Control (No IR). dH_2_O and MnCl_2_ solutions were pre-conditioned by purging with O_2_-free N_2_ (anaerobic).
(B) As for (A), but exposed to IR (10 kGy).
(C) O_2_/H_2_O_2_ was not detected in irradiated (10 kGy) TGY solid medium. After irradiation of TGY agar under O_2_-free N_2_, anaerobic dH_2_O was equilibrated in the agar tubes for 24 h before testing with the ICA and Rhodazine D assay (RDA).
(D) RDA. IR-dependent O_2_/H_2_O_2_ formation in irradiated 2 mM MnCl_2_ solutions (anaerobic).
(E) ICA. IR-dependent O_2_/H_2_O_2_ formation in irradiated MnCl_2_ solutions (anaerobic).
(F) As for (E), but O_2_/H_2_O_2_ determined by the RDA.
(G) Reference ICA color standards. A sample tube is shown: 10 mM MnCl_2_ + IR (10 kGy).
(H) Reference RDA color standards.
Theoretical rate constants for primary radiolytic reactions (The Radiation Chemistry Data Center: http://www.rcdc.nd.edu/RCDC/RCDC.html) involving the formation and decomposition of ROS in anaerobic dH_2_O do not favor significant accumulation of H_2_O_2_ or O_2_, which we did not detect in irradiated anaerobic dH_2_O or dilute MnCl_2_ solutions (<0.1 mM) ([E] and [F]).
(289 KB PDF)

Figure S2 Oxidation of Mn(II) during γ-Radiolysis of Water (^60^Co, 0 °C) Column I. 1 M MnCl_2_, no IR (aerobic); II. 1 M MnCl_2_ + 10 kGy (aerobic) (30 min after IR). Red-brown color indicates Mn(III); III. 1 M MnCl_2_ + 10 kGy (aerobic) (24 h after IR). Black color indicates Mn(IV); IV. 10 mM MnCl_2_ + 10 kGy (aerobic) (24 h after IR); and V. 1 M MnCl_2_ + 10 kGy (anaerobic, purged with Ar before IR) (24 h after IR). Notably, no Mn dioxides were precipitated during irradiation when 1 M MnCl_2_ was purged continuously with Ar (unpublished data), which further supports the idea that HO^•^ do not readily react with Mn(II) (Figure 2A; main text).
(138 KB PDF)

Figure S3 LM, TEM, and XRF Element Distribution Maps of D. radiodurans
(A) LM showing TEM grid-locations of diplococcus (group of two cells) no. 109 and no. 110.
(B) No. 109. (C) No. 110. D. radiodurans (ATCC BAA-816) diplococci were harvested from a mid-logarithmic culture (OD_600_ 0.3) in TGY. The element distribution images ([B] and [C]) are plotted to different scales designated by a single-color box, where yellow represents the highest concentration and black the lowest; ppm values in parentheses next to the element symbol correspond to yellow. For example, in (B), yellow corresponds to 200 ppm for the Mn map, but only 80 ppm for the Fe map.
(699 KB PDF)

## Abbreviations

D_10_: 10% survival value
dH_2_O: double-distilled and de-ionized water
DSB: double-strand break
HO^•^: hydroxyl radical
HO_2_^•^: hydroperoxyl radical
IR: ionizing radiation
LM: light microscopy
O_2_^• −^: superoxide ions
OD_600_: optical density at 600 nm
PolA: DNA polymerase I
ppm: parts per million
ROS: reactive oxygen species
TEM: transmission electron microscopy
XANES: X-ray-absorption near-edge structure
XRF: X-ray fluorescence
