# Supplementary material for: Protein Oxidation Implicated as the Primary Determinant of Bacterial Radioresistance
Source: PLoS Biol. 2007 Mar 20;5(4):e92. doi: 10.1371/journal.pbio.0050092 (PMC1828145; doi:10.1371/journal.pbio.0050092)
Supplement: Supplementary file 1 [file pbio.0050092.sg001.pdf]

Figure S1

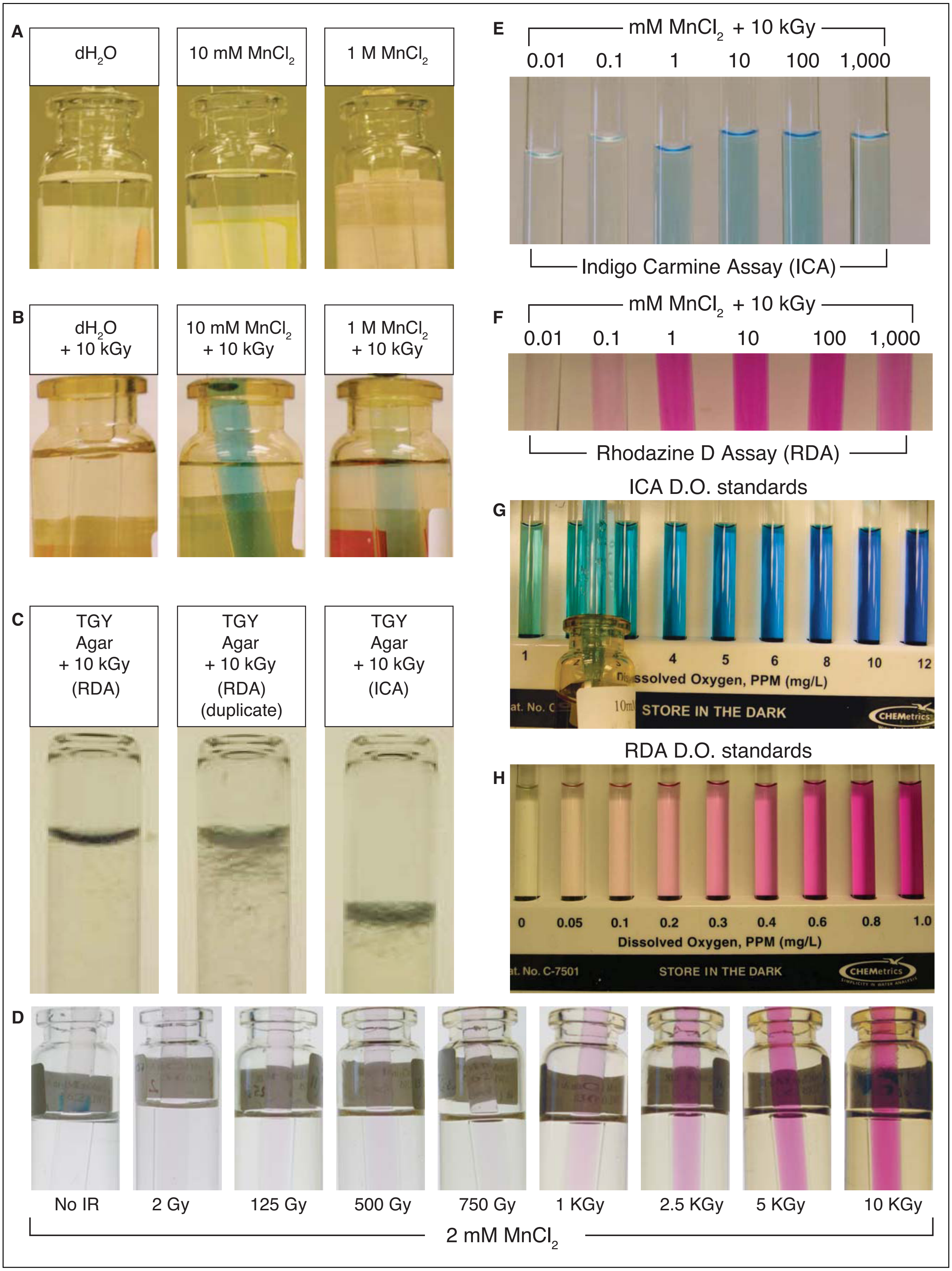
A
dH2O
10 mM MnCl2
1 M MnCl2
B
dH2O + 10 kGy
10 mM MnCl2 + 10 kGy
1 M MnCl2 + 10 kGy
C
TGY Agar + 10 kGy (RDA)
TGY Agar + 10 kGy (RDA) (duplicate)
TGY Agar + 10 kGy (ICA)
D
No IR
2 Gy
125 Gy
500 Gy
750 Gy
1 KGy
2.5 KGy
5 KGy
10 KGy
2 mM MnCl2
E
mM MnCl2 + 10 kGy
0.01
0.1
1
10
100
1,000
Indigo Carmine Assay (ICA)
F
mM MnCl2 + 10 kGy
0.01
0.1
1
10
100
1,000
Rhodazine D Assay (RDA)
ICA D.O. standards
G
1
4
5
6
8
10
12
Dissolved Oxygen, PPM (mg/L)
STORE IN THE DARK
CHEMetrics
RDA D.O. standards
H
0
0.05
0.1
0.2
0.3
0.4
0.6
0.8
1.0
Dissolved Oxygen, PPM (mg/L)
Cat. No. C-7501
STORE IN THE DARK
CHEMetrics
