# Supplementary material for: Protein Oxidation Implicated as the Primary Determinant of Bacterial Radioresistance
Source: PLoS Biol. 2007 Mar 20;5(4):e92. doi: 10.1371/journal.pbio.0050092 (PMC1828145; doi:10.1371/journal.pbio.0050092)

Figure S2

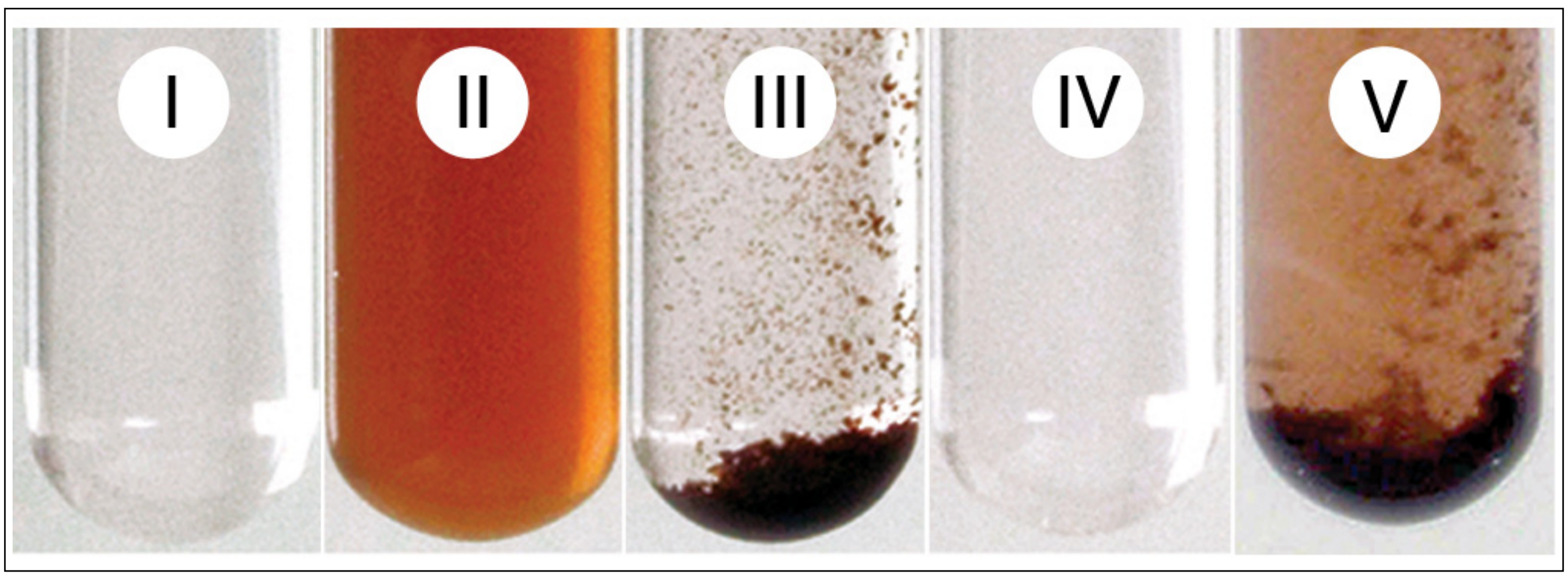

Supplement: Supplementary file 2 [file pbio.0050092.sg002.pdf]
