# Supplementary material for: Protein Oxidation Implicated as the Primary Determinant of Bacterial Radioresistance
Source: PLoS Biol. 2007 Mar 20;5(4):e92. doi: 10.1371/journal.pbio.0050092 (PMC1828145; doi:10.1371/journal.pbio.0050092)
Supplement: Supplementary file 3 [file pbio.0050092.sg003.pdf]

Figure S3

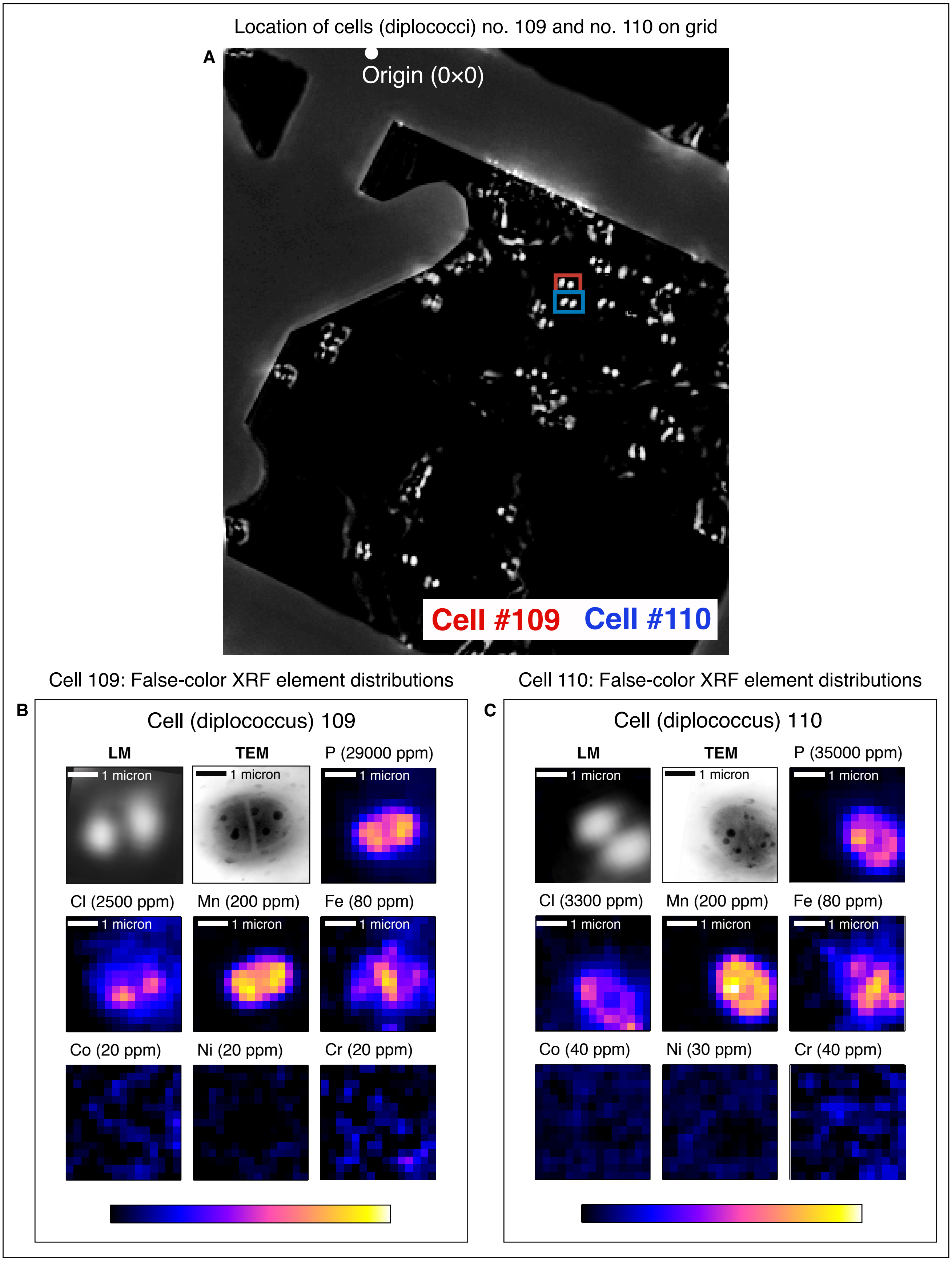
Location of cells (diplococci) no. 109 and no. 110 on grid
A
Origin (0×0)
Cell #109
Cell #110
Cell 109: False-color XRF element distributions
Cell 110: False-color XRF element distributions
B
Cell (diplococcus) 109
LM
TEM
P (29000 ppm)
1 micron
Cl (2500 ppm)
Mn (200 ppm)
Fe (80 ppm)
Co (20 ppm)
Ni (20 ppm)
Cr (20 ppm)
C
Cell (diplococcus) 110
LM
TEM
P (35000 ppm)
Cl (3300 ppm)
Mn (200 ppm)
Fe (80 ppm)
Co (40 ppm)
Ni (30 ppm)
Cr (40 ppm)
